# 

**Published:** 1896-12

**Authors:** 


### CONTENTS.

ORIGINAL COMMUNICATIONS—

THE HISTORY AND INTRODUCTION OF ANESTHESIA IN SURGERY. By Roswell Park, A.M., M.D., Buffalo, N. Y. .... 649

THE TREATMENT OF SPRAINED ANKLE. By W. S. Elkin, M.D., Atlanta, Ga. .... 662

DISEASES OF THE RECTUM AND ANUS. By Geo. K. Sims, M.D., Richmond, Va. .... 664

THREE CASES OF POST-TYPHOID NEURITIS. By Geo. J. Preston, M.D., Baltimore, Md. 672

SYSTEMATIC INSPECTION OF THE EYE. By H. E. Stafford, M.D., New York .... 674

SOME REMARKS ON THE THERAPEUTICS OF FERRATIN AND LACTOPHENIN. By T. V. Hubbard, M.D., Atlanta, Ga. .... 677

SOCIETY REPORTS—

ATLANTA SOCIETY OF MEDICINE .... 681

TRI-STATE MEDICAL SOCIETY OF ALABAMA, GEORGIA AND TENNESSEE .... 687

CORRESPONDENCE—

THE BOARD OF EXAMINERS AND THE ATLANTA CLINIC .... 695

MY FIRST CASE OF PUERPERAL CONVULSIONS .... 698

EDITORIAL—

THE SEMI-CENTENNIAL OF THE DISCOVERY OF ANESTHESIA .... 701

A MEDICAL DEGREE FOR THIRTY-FIVE DOLLARS .... 702

THE EXAMINING BOARD AND ITS CRITICS .... 703

SELECTIONS AND ABSTRACTS .... 704

MEDICAL ITEMS .... 716

BOOK REVIEWS .... 718

MEDICAL ITEMS—CONTINUED .... x, xii, xiii, xiv, xv

ADVERTISERS' INDEX TO ADVERTISEMENTS .... xvi

CLASSIFIED INDEX TO ADVERTISEMENTS .... xxxiii

